# Applications of Frameless Image-Guided Robotic Stereotactic Radiotherapy and Radiosurgery in Pediatric Neuro-Oncology: A Systematic Review

**DOI:** 10.3390/cancers14041085

**Published:** 2022-02-21

**Authors:** Felix Ehret, David Kaul, Volker Budach, Laura-Nanna Lohkamp

**Affiliations:** 1 Berlin Institute of Health at Charité—Universitätsmedizin Berlin, 10117 Berlin, Germany; 2 Charité—Universitätsmedizin Berlin, Corporate Member of Freie Universität Berlin and Humboldt-Universität zu Berlin, Department of Radiation Oncology, 13353 Berlin, Germany; david.kaul@charite.de (D.K.); volker.budach@charite.de (V.B.); 3 European Radiosurgery Center, 81377 Munich, Germany; 4 Charité—Universitätsmedizin Berlin, Corporate Member of Freie Universität Berlin and Humboldt-Universität zu Berlin, Charité CyberKnife Center, 13353 Berlin, Germany; 5 German Cancer Consortium (DKTK), Partner Site Berlin, German Cancer Research Center (DKFZ), 69120 Heidelberg, Germany; 6 Division of Neurosurgery, Department of Surgery, Toronto Western Hospital, University Health Network, University of Toronto, Toronto, ON M5T 2S8, Canada; laura-nanna.lohkamp@mail.de

**Keywords:** pediatric neuro-oncology, neuro-oncology, systematic review, PRISMA, stereotactic radiosurgery, stereotactic radiotherapy, robotic radiosurgery, CyberKnife

## Abstract

**Simple Summary:**

Radiotherapy plays a vital role in the multimodal treatment of pediatric central nervous system (CNS) tumors. In cases of small-to-medium-sized, well-demarcated lesions, high-precision treatment modalities such as stereotactic radiosurgery (SRS) are well-established in adult patients. SRS and, more specifically, robotic radiosurgery (RRS) and robotic stereotactic radiotherapy (RSRT) have only limited evidence in the field of pediatric neuro-oncology. This systematic review aims to report and assess the available RRS and RSRT data and studies. Results demonstrate that both treatment modalities are infrequently applied and primarily used in specific situations, including postoperative, palliative, and salvage treatments. Treatment outcomes are encouraging, but high-quality studies are lacking. Prospective studies are necessary to determine the actual utility of RRS and RSRT in pediatric neuro-oncology. Nevertheless, RRS and RSRT may be applied for selected patients.

**Abstract:**

Background: CyberKnife-based robotic radiosurgery (RRS) is a widely used treatment modality for various benign and malignant tumors of the central nervous system (CNS) in adults due to its high precision, favorable safety profile, and efficacy. Although RRS is emerging in pediatric neuro-oncology, scientific evidence for treatment indications, treatment parameters, and patient outcomes is scarce. This systematic review summarizes the current experience and evidence for RRS and robotic stereotactic radiotherapy (RSRT) in pediatric neuro-oncology. Methods: We performed a systematic review based on the databases Ovid Medline, Embase, Cochrane Library, and PubMed to identify studies and published articles reporting on RRS and RSRT treatments in pediatric neuro-oncology. The Preferred Reporting Items for Systematic Reviews and Meta-Analyses (PRISMA) guidelines were applied herein. Articles were included if they described the application of RRS and RSRT in pediatric neuro-oncological patients. The quality of the articles was assessed based on their evidence level and their risk for bias using the original as well as an adapted version of the Newcastle Ottawa Quality Assessment Scale (NOS). Only articles published until 1 August 2021, were included. Results: A total of 23 articles were included after final review and removal of duplicates. Articles reported on a broad variety of CNS entities with various treatment indications. A majority of publications lacked substantial sample sizes and a prospective study design. Several reports included adult patients, thereby limiting the possibility of data extraction and analysis of pediatric patients. RRS and RSRT were mostly used in the setting of adjuvant, palliative, and salvage treatments with decent local control rates and acceptable short-to-intermediate-term toxicity. However, follow-up durations were limited. The evidence level was IV for all studies; the NOS score ranged between four and six, while the overall risk of bias was moderate to low. Conclusion: Publications on RRS and RSRT and their application in pediatric neuro-oncology are rare and lack high-quality evidence with respect to entity-related treatment standards and long-term outcomes. The limited data suggest that RRS and RSRT could be efficient treatment modalities, especially for children who are unsuitable for surgical interventions, suffer from tumor recurrences, or require palliative treatments. Nevertheless, the potential short-term and long-term adverse events must be kept in mind when choosing such a treatment. Prospective studies are necessary to determine the actual utility of RRS and RSRT in pediatric neuro-oncology.

## 1. Introduction

Stereotactic radiosurgery (SRS), stereotactic radiotherapy (SRT), and stereotactic body radiotherapy (SBRT) are well-established treatment modalities for a wide range of benign and malignant tumors [1,2]. Specifically, SRS plays a crucial role in the modern management of central nervous system (CNS) tumors [1]. Treatment algorithms and outcome data are well-reported and documented in adult patients. In the field of pediatric neuro-oncology, however, analogous data for SRS and, more specifically, robotic radiosurgery (RRS) and robotic stereotactic radiotherapy (RSRT) remain scarce [3,4,5]. So far, a limited number of radiosurgical case reports and case series are available for variable neuro-oncological entities, including ependymoma, low- and high-grade glioma, medulloblastoma, meningioma, craniopharyngioma, pituitary adenoma, pineal tumors, arteriovenous malformation, and vestibular schwannoma [3,4,5,6]. Given the frameless treatment delivery, precision, and increasing availability, RRS, and RSRT may represent valuable treatment options in managing pediatric CNS tumors. This comprehensive review aims to summarize the current findings and evidence for the use of RRS and RSRT in pediatric neuro-oncology.

## 2. Materials and Methods

### 2.1. Literature Search and Review

A comprehensive literature search was performed by two independent reviewers (F.E., L.-N.L.) using the databases Ovid Medline, Embase, Cochrane Library, and PubMed according to the 2020 Preferred Reporting Items for Systematic Reviews and Meta-Analyses (PRISMA) guidelines [7]. Combinations of the search terms “CNS”, “brain”, and “radiosurgery”, complemented by “pediatric” or “children” and “image-guided robotic radiosurgery” or “CyberKnife” were applied without restrictive filters or limits. An additional citation search was performed amongst the articles that resulted from the initial database search. Both reviewers (F.E., L.-N.L.) independently conducted the screening, removed duplicates, excluded unsuitable articles, and agreed on the final selection of included publications. Removal of duplicates was done using EndNote 20.2 (EndNote, Clarivate, Philadelphia, PA, USA), followed by manual verification. Reported indications, general patient characteristics, parameters pertaining to the radiosurgical procedure, as well as clinical and radiographic outcomes, including the length of follow-up, were summarized. Articles were included if they described the application of RRS or RSRT using the CyberKnife^®^ radiosurgery system (Accuray Inc., Sunnyvale, CA, USA) for CNS tumors in children, regardless of the number of actual treatments or whether they were combined studies and included outcomes for adult patients. Only articles available in English published before 1 August 2021, were considered. This review was registered (Research Registry, Research Registry Unique Identifying Number 1258).

### 2.2. Quality and Bias Assessment

The quality of the articles was assessed by both reviewers (F.E., L.-N.L.). Articles were evaluated according to their evidence level according to the Centre for Evidence Based Medicine (CEBM) and using items of the Newcastle-Ottawa Quality Assessment (NOS) Scale in a modified version by Murad et al. [8,9,10,11,12]. The modification was made based on a study of Bazerbachi et al., given that there are no validated tools to assess the risk of bias (i.e., methodological quality) in case reports and case series, which they had taken into account [12]. The majority of the included studies herein were noncomparative case reports or case series, thereby preventing the application of NOS that are related to comparability and adjustment. According to Bazerbachi et al.’s protocol, this resulted in applying the NOS items that focused on selection, representativeness of cases, and ascertainment of outcome and exposure. Accordingly, we included five criteria in the form of questions with a binary response (yes/no), addressing whether the item was suggestive of bias or not [12]. We applied the same quality scale as previously described, considering the quality of the report good (low risk of bias) when all five criteria were fulfilled, moderate when four were fulfilled, and poor when three or fewer were fulfilled [12]. This review did not require an institutional review board approval given the local regulations and chosen study methodologies.

## 3. Results

**Figure 1:**
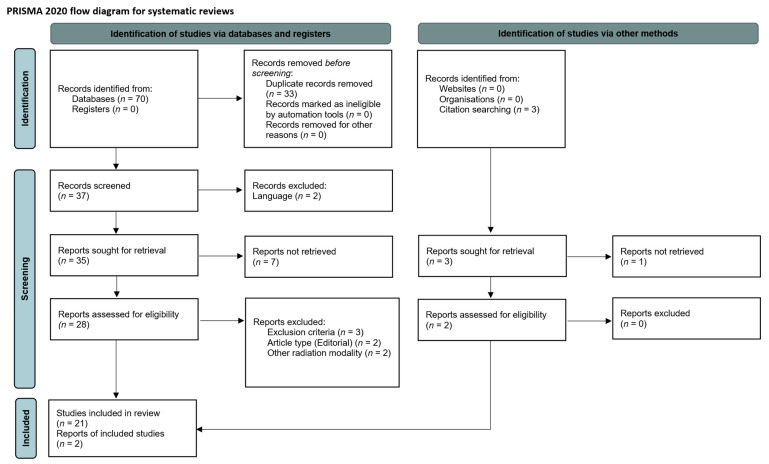
Flowchart illustrating the article selection process according to the PRISMA 2020 guidelines.

Seventy reports were initially identified during the database searches and an additional three articles by citation searching. After removing duplicates and non-English articles, screening, and further evaluation, 30 articles remained for the eligibility assessment. In the end, 23 studies were included in the review (Figure 1). The first report, a case report of a recurrent vestibular schwannoma treated with RRS in a 13-year-old boy, was published in 2000 [13]. The latest study addressed the treatment of spinal ependymomas, included two pediatric cases, and was published in 2021 [14]. The studies reviewed herein reported on a broad variety of CNS entities, including arteriovenous malformations (AVM), neuroblastoma, ependymoma, vestibular schwannoma, chordoma, craniopharyngioma (CP), fibrosarcoma, hamartoma, pineal germinoma, pituitary adenoma, optic pathway or pilocytic glioma, Ewing sarcoma, atypical teratoid rhabdoid tumor (ATRT), and medulloblastoma. Notably, most studies (12 out of 23, 52%) did not exclusively report on the use of RRS in pediatric patients, significantly limiting data extraction for the respective treatments. At least 125 patients with approximately 142 treatments were summarized in the reviewed articles. In five studies (22%), at least one pediatric patient was included without dedicated information about the actual number of patients and RRS or RSRT treatments [15,16,17,18,19]. More extensive series on RRS were scarce, and the available data was mostly limited to case reports and smaller case series (Table 1). Sample sizes of the studies varied from case reports with a single patient (*n* = 5) to clinical articles including up to 52 pediatric patients [20]. The age of treated pediatric patients ranged from four months to 23 years. The age limit in most of the dedicated pediatric studies was defined as <18 years. However, two studies included patients older than 18 years.

### 3.1. Treatment Indications, Parameters, and Outcomes

The most common indications of RRS and RSRT were recurrences, adjuvant, and salvage treatments, with primary treatments only being reported in a minority of studies. Dosimetric analyses of the studies revealed that most treatments utilized RRS, with up to five fractions. However, the largest series of 52 patients of Mohamad et al. applied RSRT using 25 to 33 fractions to irradiate craniopharyngiomas, ependymomas, and low-grade gliomas [20]. In this study, margin-free RSRT was mostly used for adjuvant treatments in a pediatric patient cohort with well-demarcated brain tumors. Thirty representative cases were also planned with conventional intensity-modulated RT (IMRT) with subsequent comparison of the dosimetric plans. RSRT plans showed superior dosimetry with a significant reduction in both the high and intermediate dose regions. Concerning the clinical and radiographic outcomes, a 3-year local control (LC) of 92% was achieved. The observed toxicity was limited to transient perilesional edema in 33% of patients, whereas 7 of 16 CP patients suffered from cystic lesion enlargement requiring further interventions [20]. Another extensive report was published in 2005 by Giller et al., describing outcomes of 38 treatments in 21 pediatric patients with unresectable brain tumors [21]. Irradiated entities included astrocytoma, ependymoma, medulloblastoma, ATRT, and craniopharyngioma [21]. This series also showed a decent LC in all reported entities, with most treatments delivered in just one session [21]. In four patients, who were aggressively treated due to their progressive disease, radionecrosis was observed, with one patient becoming symptomatic [21]. This report did not describe any further adverse events.

The case reports summarized herein are comparable in terms of tumor entities, indication, and dosimetric parameters to the larger reports of Mohamad et al. and Giller et al., reporting on very specific, individual treatments [20,21]. For example, Romanelli reported two cases of pediatric patients suffering from unresectable hypothalamic hamartomas causing severe, daily gelastic, and generalized tonic-clonic seizures [22]. Despite intensive usage of up to five antiepileptic drugs, seizure control remained poor [22]. In an attempt to reduce seizure frequency and severity, the eight- and nine-year-old children underwent single-session RRS. Eighteen and 36 months after treatment, respectively, both patients started to remain seizure-free for the available follow-up of 10 and 9 years [22]. Discontinuation of antiepileptic medication started two and three years after RRS, respectively [22]. No treatment-related toxicity was observed. This is one of the available case reports and series demonstrating the potential efficacy and safety of RRS in selected patients.

**Table 1:** Table summarizing the current literature on RRS and RSRT in pediatric CNS pathologies, indicating the number of pediatric patients reported, diagnosis, treatment indication and parameters as well as their clinical and radiographic outcome. Abbreviations: ATRT (atypical teratoid rhabdoid tumor), AVM (arteriovenous malformation), DF (distant failure), EP (ependymoma), EW (Ewing sarcoma), f (female), FU (follow-up), Gy (Gray), LF (local failure), m (male), MB (medulloblastoma), n.a. (not assessed/assessable), LGG (low grade glioma), OPG (optic pathway glioma), TX (therapy), cc (cubic centimeters). * Publication including only pediatric patients.

Author	Year	Number of pediatric patients (total patients)	Age (years)	Gender	Tumor entity	Treatment indication	Mean FU (months)	Mean survival (months)	Dose (Gy)	Fractions (n)	Prescription isodose line (%)	Volume (cc)	Clinical outcome	Radiographic outcome
Ehret et al. [14]	2021	2 (12)	<18	n.a.	Spinal ependymoma	Adjuvant TX; recurrence	n.a.	n.a.	n.a.	n.a.	n.a.	n.a.	n.a.	n.a.
Mohamad et al. * [20]	2020	52	9.9 (range 1.1–23.2)	f (23); m (29)	Craniopharyngioma; ependymoma; LGG	Primary TX; adjuvant TX	44.4	36 (OS 100% at 3 years)	45 to 60	25–33	84 (range 52–91)	10.3 (1.1–38.1)	n.a.	Resolution /decrease (37); stable tumor (14); tumor progression (1)
Shi et al. * [23]	2019	11 (21)	3 (mean); range 0–19	n.a.	Intracranial and spinal ependymomas	Adjuvant; salvage TX	54 median (range 2–157)	n.a	18–20	1–5	n.a.	n.a.	In children: Radiation toxicity (2), death (1)	Overall LF after 2-years 18.5%; DF after 2 years 33.8%
Fadel et al. [24]	2019	2	8; 10	m (1); f (1)	Intracranial oculomotor nerve schwannomas	Primary TX	57	n.a.	45–50	25	n.a.	0.1; 0.2	Neurologically stable	Decrease in tumor volume (1)
Romanelli et al. * [22]	2018	2	8; 9	n.a.	Hypothalamic hamartoma	Refractory medical TX	36; 42	n.a.	16 (max dose 24.43; 22.85)	1	65; 70	1.1; 0.89	Seizure freedom at last FU	Transient focal edema
Kalani et al. [19]	2016	≥1 (37)	≥9	n.a.	Spinal cord arteriovenous malformations	Primary TX; second line TX	n.a.	n.a.	n.a.	n.a.	n.a.	n.a.	n.a	n.a.
Nanda et al. * [25]	2014	5	5.7 (range 2.7–11.3)	n.a.	EP (2), MB (1); ATRT (1); EW (1)	Recurrence; palliative TX	22.8 (range 1–45)	22.8 (range 1–45)	15–21	4.4 (range 1–10)	n.a.	0.08 to 51.67	Alive (2); death (3)	TX failure: in field (3); distant (2)
Susheela et al. * [26]	2013	1	12	m (1); f (0)	Hypothalamic hamartoma	Refractory medical TX	17	n.a.	30	5	85	48.3	Seizure freedom	Transient focal edema
Uslu et al. * [27]	2013	1	11	m (0); f (1)	OPG	Primary TX	17	n.a.	21	5	83	5.2	Minimal radiation effects: conjunctivitis, dry eyes	Decrease of tumor volume
Lo et al. * [28]	2013	1	8 months	m	Infantile fibrosarcoma spinal metastasis	Concomitant with CTX	33	n.a.	26	4	75	8.8	Stable	Tumor size reduction of 23%
Iwata et al. [18]	2012	≥1 (43)	≥3	n.a.	Craniopharyngioma	Inoperabilty; adjuvant TX; recurrence	n.a.	n.a.	n.a.	n.a.	n.a.	n.a.	n.a.	n.a.
Jiang et al. [29]	2012	3 (20)	10; 12; 17	m (3); f (0)	Chordoma	Recurrence	21.3	n.a.	30; 25; 37.5	5 (3)	75; 80; 80	17.4; 10.4; 2.4	Death (2); neurological improvement (1)	Tumor size reduction (1)
Chen et al. * [30]	2012	1	3	m (1); f (0)	Neuroblastoma metastasis	New intracranial metastasis	6	n.a	21	5	n.a.	n.a.	Neurological and hearing improvement	Decrease of tumor size
Peugniez et al. * [31]	2010	5	8.2 (range 8–10)	f (1); m (4)	OPG (2); Pineal germinoma (1); MB (1); EW (1)	Residual; recurrence	8.6 (range 6–12)	n.a.	36.4 (range 19.8–50.4)	22.8 (range 11–28)	n.a.	n.a.	Stable disease (3); progressive disease (2)	n.a.
Colombo et al. [32]	2009	2 (279)	12; 12	m (0); f (2)	AVM	not specified	n.a.	n.a.	24; 25	1	n.a.	2.2; 2.8	n.a.	n.a.
Coppa et al. [15]	2009	≥1 (31)	11	n.a.	Malignant skull base tumors	Recurrence; inoperability	n.a.	n.a.	n.a.	n.a.	n.a.	n.a.	n.a.	n.a.
Gagnon et al. [17]	2009	≥1 (200)	3	n.a.	Benign and malignant spinal tumors	Primary TX; adjuvant TX; recurrence	n.a.	n.a.	n.a.	n.a.	n.a.	n.a.	n.a.	n.a.
Lee et al. [33]	2008	3 (11)	13; 16; 17	m (0); f (3)	Craniopharyngioma	Residual; recurrence	n.a.	n.a.	19.5; 20; 27.5	3; 4; 5	80; 77; 71	12.7; 1.2; 10.1	Stable	n.a.
Dodd et al. [16]	2006	≥1 (51)	12	n.a.	Benign spine tumors	Recurrence; residual tumor; inoperability	n.a.	n.a.	n.a.	n.a.	n.a.	n.a.	n.a.	n.a.
Giller et al. * [21]	2005	21 (38 treatments)	8 months to 16 years (mean 7± 5.1 years; median 6 years)	m (8); f (13)	Various brain tumors	Inoperability; unresponsiveness to standard TX; focal recurrence or residual	18 ± 11 (range 1–40)	21 ± 11 (range 1–40)	18.8 ± 8.1 (range 9.2–50, median 17)	1 (27); 3–5 (8); conventional (3)	57 ± 9.7 (range 35–90; median 60)	10.7 ± 20 (range 0.06–103)	Death (6)	Reported per entity
Kajiwara et al. [34]	2005	2 (21)	11; 11	m (0); f (2)	Pituitary adenoma	Second line TX	55	n.a.	9.44; 27	3	n.a.	7.0; 0.2	Hypopituitarism (1)	Partial response; no change
Giller et al. * [35]	2004	5	4 months; 7 months; 11 months; 1 year; 2.5 years;	m (1); f (4)	Malignant brain tumors	Salvage TX; concomitant	11 ± 7 (range 5–23)	n.a.	17 ± 2 (range 15 – 24)	1 (1), 4 (1), 5 (3)	45 to 65	18 ± 22	Death (2)	Decrease in lesional size (2), local recurrence (1), distant metastasis (2)
Harada et al. * [13]	2000	1	13	m	Acoustic schwannoma	Salvage TX after 3rd recurrence	n.a.	n.a.	n.a.	n.a.	n.a.	15	n.a.	n.a.

In summary, applied doses, used prescription isodose lines, and fractions among the reviewed studies herein were particularly heterogeneous (Table 1). This was also the case for the treated tumor volumes, given the various indications and tumor entities. However, the reported clinical and radiographical outcomes were equally rated as favorable in the majority of the case reports. A detailed overview of the included studies is provided in Table 1.

### 3.2. Quality and Bias Assessment

The quality of the articles was assessed according to their level of evidence developed by the CEBM for treatment and with the NOS, when applicable. All reviewed studies provided scientific reporting on an evidence level of IV. We considered 13 articles eligible for the NOS based on their patient numbers, excluding case reports and smaller case series. In these studies, the NOS ranged between four and six stars, including nine articles receiving six (69%), three receiving five (23%), and one receiving four stars (8%), respectively. Notably, none of the studies were eligible for stars in the comparability section as they lacked control groups or cohorts. Therefore, an additional rating system, which was proposed as a modified version of the NOS, was applied for assessing the risk of bias in the included studies (Table 2) [12]. Fourteen out of 23 studies had a low risk of bias, followed by seven with a moderate and two with a high risk. The main sources for introducing bias were the exclusion of differential diagnosis, the lack of data citation, and comprehensive outcome reporting (Table 2).

## 4. Discussion

Radiotherapy plays an essential role in the treatment of various pediatric brain tumors [36,37,38,39]. As a specific subtype of radiotherapy, SRS can deliver highly conformal irradiations with steep dose gradients, ultimately preventing organs at risk and healthy tissue from radiation [40]. Notably, RRS as a frameless, image-guided radiosurgical treatment technique may effectively reduce high-dose exposure to adjacent healthy tissue [41]. However, its application has mainly been investigated and reported in adult neuro-oncology. Despite its benefits in adult patients, data, studies, and clinical trials for SRS and, more specifically, RRS as well as RSRT, in pediatric patients remain scarce [3,4].

This systematic review had the objective to assess the current literature on RRS and RSRT in pediatric neuro-oncological patients, with a particular focus on treated tumor entities, fractionation schemes, and outcomes. Given the general data available on SRS for pediatric brain tumors, a significant degree of data heterogeneity and overall limited sample sizes are apparent [3,4]. These findings were also confirmed for RRS in the review herein: radiosurgical treatments are mostly used as an adjunct in the multimodal treatment of brain tumors. Its applications are currently reserved for adjuvant, salvage, and palliative treatments, as well as for unresectable tumors or tumor remnants. Despite the data heterogeneity and small cohorts, included RRS and RSRT studies showed favorable results for the various reported neuro-oncological treatments. Lo et al. and Murphy et al. already addressed various advantages and disadvantages of SRS in the setting of pediatric neuro-oncology [3,4]. In general, the dosimetric advantages and time-saving treatment delivery of SRS are particularly helpful treating well-demarcated lesions. This also applies to RRS [20,21]. Moreover, the non-invasive, frameless treatment technique of RRS and other SRS techniques is another aspect of highest importance, especially in children, as it does not require rigid fixation, which subsequently allows a reduction of general anesthesia procedures compared to other radiation techniques [4,21,25,35,42]. This setting frames the potential role of SRS, including RRS, in the management of pediatric brain tumors. Entities and lesions such as vestibular schwannomas, pituitary adenomas, meningiomas, neurocytoma, and small metastases may be effectively addressed with this treatment modality [3,4,21,35,43]. Similar to adults, recent data also suggest AVMs to be a suitable pediatric SRS target [6,44]. In contrast, diffuse tumor growth usually dictates other treatment options, such as surgery or fractionated radiotherapy. The latter may also be delivered utilizing RSRT based on the large series of Mohamad et al., showing promising results and favorable dosimetric profiles compared to conventional IMRT [20]. However, only well-demarcated targets have been included in this analysis. Nevertheless, these first hypothesis-generating results open room for further investigations utilizing the highly conformal treatments with RSRT in children. Prospectively comparing those findings to IMRT or proton radiotherapy could help to refine neuro-oncological treatments and identify potential patient cohorts or entities, which profit from such treatment techniques [45].

In this regard, treatment-related complications and adverse events (AE) play a crucial role in children, especially when evaluating an emerging radiation technique [46]. Numerous studies have demonstrated the manifold short-term and long-term complications of CNS irradiations in pediatric patients, ranging from symptomatic radiation necrosis, cognitive deficits, growth abnormalities, and endocrinological disorders to secondary malignancies in survivors [21,39,47,48,49]. Potential approaches to prevent such sequelae include the reduction of treatment volumes, margins, and applied doses [39]. While the first two points may be efficiently implemented with RRS due to the underlying dosimetric characteristics, the latter remains the subject of further investigations, especially for fractionated radiotherapy or fractionated stereotactic radiotherapy, whereas in SRS, locally ablative doses are an essential part of the treatment modality [39]. Therefore, the risk for radiation necrosis should be considered when choosing the radiation treatment modality [21]. Carefully balancing treatment aggressiveness with the risk of potentially devastating adverse events depending on the patients’ disease, life expectancy, and performance status is a crucial objective in pediatric neuro-oncology and is mainly guided by entity-specific treatment protocols and interdisciplinary tumor boards.

Considering the current evidence for RRS and RSRT, we suggest their application in selected pediatric patients and in accordance with the local treatment protocols or after agreement with an interdisciplinary neuro-oncological tumor board. Furthermore, treatments should be carried out by experienced radiation oncologists or neurosurgeons and include a dedicated long-term follow-up with repeated neuropsychological testing and imaging. To enhance the availability of data concerning the efficacy and safety of RRS and RSRT in pediatric neuro-oncology, a national registry was recently initiated at our institution [50]. Given the imbalance of scientific evidence between adult and pediatric RRS and RSRT treatments, it aims to fill the current knowledge gaps on the pediatric side, hoping that the obtained results may contribute to the quality improvement of radiosurgical treatments in pediatric neuro-oncology. In summary, RRS represents a viable and versatile tool for the treatment of brain and spine lesions. However, the evidence for its usage in pediatric neuro-oncology remains limited and on a descriptive level. This is highlighted by the lack of randomized trials and low evidence levels of available analyses. Current reports lack the sample sizes, comparability, and standardization to draw firm conclusions. The articles demonstrated a moderate to low risk of bias and showed encouraging outcome results and confirmation of tolerability and feasibility in children. Collaborative efforts are necessary to determine the potential role of RRS and RSRT in pediatric neuro-oncology.

## 5. Conclusions

RRS and its applications in pediatric neuro-oncology have rarely been reported thus far. The limited data suggest that RRS and RSRT could be efficient treatment modalities, especially for children who are unsuitable for surgical interventions, suffer from tumor recurrences, or require palliative treatments. Nevertheless, the potential short-term and long-term adverse events must be kept in mind when choosing such a treatment. Further studies of prospective nature are necessary to determine the actual utility and safety profile of RRS and RSRT in pediatric neuro-oncology.

## Figures and Tables

**Table 2:** Table illustrating the current literature on RRS and RSRT in pediatric CNS pathologies, including the article type, the level of evidence, as well as the quality based on the original and modified Newcastle-Ottawa Quality Assessment Scale NOS) [12]. Questions 1–5 comprise the tool for risk of bias assessment for case reports and case series: (1) Did the patient(s) represent the whole case(s) of the medical center? (The studies did not mention whether the reported patient(s) represented the whole case(s) of the medical center and we assumed that the authors have reported all the cases in their center giving the rarity of this association.) (2) Was the diagnosis correctly made? (3) Were other important diagnoses excluded? (4) Were all important data cited in the report? (5) Was the outcome correctly ascertained?

Author	Year	Article type	Evidence level	NOS Scale				Modified NOS					
				Selection (★/4)	Comparability (★/2)	Exposure/Outcome (★/3)	Total (9★)	Completeness	Correct diagnosis	Differential Diagnosis	Citation of data	Outcome	Risk of bias
Ehret et al.	2021	Research article	IV	3/4	0/2	3/3	6/9	yes	yes	yes	yes	yes	low
Mohamed et al. *	2020	Original article	IV	3/4	0/2	3/3	6/9	yes	yes	yes	yes	yes	low
Shi et al. *	2019	Clinical article	IV	3/4	0/2	3/3	6/9	yes	yes	yes	yes	yes	low
Fadel et al.	2019	Literature review with case series	IV	n.a.	n.a.	n.a.	n.a.	yes	yes (presumed)	no	yes	yes	moderate
Romanelli et al. *	2018	Case series	IV	n.a.	n.a.	n.a.	n.a.	yes	yes	yes	yes	yes	low
Kalani et al.	2016	Clinical article	IV	2/4	0/2	3/3	5/9	yes	yes	yes	yes	yes	low
Nanda et al. *	2014	Case series	IV	n.a.	n.a.	n.a.	n.a.	yes (presumed)	yes	yes	yes	yes	low
Susheela et al. *	2013	Case report	IV	n.a.	n.a.	n.a.	n.a.	yes	yes	yes	yes	yes	low
Uslu et al. *	2013	Case report	IV	n.a.	n.a.	n.a.	n.a.	yes	yes (presumed)	no	yes	yes	moderate
Lo et al. *	2013	Case report	IV	n.a.	n.a.	n.a.	n.a.	yes	yes	yes	yes	yes	low
Iwata et al.	2012	Clinical article	IV	3/4	0/2	3/3	6/9	yes	yes	yes	yes	yes	low
Jiang et al.	2012	Clinical article	IV	3/4	0/2	3/3	6/9	yes	yes	yes	yes	yes	low
Chen et al. *	2012	Case report	IV	n.a.	n.a.	n.a.	n.a.	yes	yes (presumed)	no	yes	yes	moderate
Peugniez et al. *	2010	Case series	IV	n.a.	n.a.	n.a.	n.a.	yes	yes	yes	no	no	high
Colombo et al.	2009	Clinical article	IV	3/4	0/2	3/3	6/9	no	yes	yes	yes	yes	low
Coppa et al.	2009	Research article	IV	3/4	0/2	3/3	6/9	yes	yes	yes	yes	yes	low
Gagnon et al.	2009	Clinical article	IV	3/4	0/2	3/3	6/9	yes	yes	yes	yes	yes	low
Lee et al.	2008	Clinical article	IV	3/4	0/2	1/3	4/9	yes	yes	yes	no	yes	moderate
Dodd et al.	2006	Clinical article	IV	3/4	0/2	2/3	5/9	yes	yes (presumed)	no	yes	yes	moderate
Giller et al. *	2005	Clinical article	IV	2/4	0/2	3/3	5/9	yes	yes	yes	no	yes	moderate
Kajiwara et al.	2005	Clinical article	IV	3/4	0/2	3/3	6/9	yes	yes	yes	yes	yes	low
Giller et al. *	2004	Technical report with case series	IV	n.a.	n.a.	n.a.	n.a.	yes (presumed)	yes	yes	no	yes	moderate
Harada et al.	2000	Case report	IV	n.a.	n.a.	n.a.	n.a.	yes	yes	yes	no	no	high
* Publication including only pediatric patients.													
